# SCMBYK: prediction and characterization of bacterial tyrosine-kinases based on propensity scores of dipeptides

**DOI:** 10.1186/s12859-016-1371-4

**Published:** 2016-12-22

**Authors:** Tamara Vasylenko, Yi-Fan Liou, Po-Chin Chiou, Hsiao-Wei Chu, Yung-Sung Lai, Yu-Ling Chou, Hui-Ling Huang, Shinn-Ying Ho

**Affiliations:** 1 Institute of Bioinformatics and Systems Biology, National Chiao Tung University, Hsinchu, 300 Taiwan; 2 College of Biological Science and Technology, National Chiao Tung University, Hsinchu, 300 Taiwan; 3 Center for Bioinformatics Research, National Chiao Tung University, Hsinchu, Taiwan

**Keywords:** BY-kinase, Scoring card method, Drug repurposing, Propensity scores, Dipeptide

## Abstract

**Background:**

Bacterial tyrosine-kinases (BY-kinases), which play an important role in numerous cellular processes, are characterized as a separate class of enzymes and share no structural similarity with their eukaryotic counterparts. However, *in silico* methods for predicting BY-kinases have not been developed yet. Since these enzymes are involved in key regulatory processes, and are promising targets for anti-bacterial drug design, it is desirable to develop a simple and easily interpretable predictor to gain new insights into bacterial tyrosine phosphorylation. This study proposes a novel SCMBYK method for predicting and characterizing BY-kinases.

**Results:**

A dataset consisting of 797 BY-kinases and 783 non-BY-kinases was established to design the SCMBYK predictor, which achieved training and test accuracies of 97.55 and 96.73%, respectively. Furthermore, the leave-one-phylum-out method was used to predict specific bacterial phyla hosts of target sequences, gaining 97.39% average test accuracy. After analyzing SCMBYK-derived propensity scores, four characteristics of BY-kinases were determined: 1) BY-kinases tend to be composed of α-helices; 2) the amino-acid content of extracellular regions of BY-kinases is expected to be dominated by residues such as Val, Ile, Phe and Tyr; 3) BY-kinases structurally resemble nuclear proteins; 4) different domains play different roles in triggering BY-kinase activity.

**Conclusions:**

The SCMBYK predictor is an effective method for identification of possible BY-kinases. Furthermore, it can be used as a part of a novel drug repurposing method, which recognizes putative BY-kinases and matches them to approved drugs. Among other results, our analysis revealed that azathioprine could suppress the virulence of *M. tuberculosis*, and thus be considered as a potential antibiotic for tuberculosis treatment.

**Electronic supplementary material:**

The online version of this article (doi:10.1186/s12859-016-1371-4) contains supplementary material, which is available to authorized users.

## Background

Bacterial tyrosine-kinases (BY-kinases) are enzymes that perform protein phosphorylation and autophosphorylation, and have been identified in the majority of sequenced bacterial genomes [1–3]. They transfer phosphate groups from ATP to reactive side chains of Tyr residues, regulating processes of cellular signaling [3]. BY-kinases have been shown to have no resemblance with their counterparts in *Eukarya*, and have been classified as a separate protein family [1, 2].

A typical BY-kinase contains two domains: a transmembrane activator domain (TAD) that includes a large extracellular loop, and an intracellular catalytic domain (CD) [2, 3]. These domains are either encoded by a single gene and are parts of the same protein (e.g., in *Escherichia coli*), or are encoded by two adjacent genes and exist as two proteins: one transmembrane and another cytoplasmic protein (e.g., in *Bacillus subtilis*). The CD domain performs the phosphorylation of tyrosine, while the intracellular juxtamembrane region of the TAD is essential for the activation of the CD domain [3]. The CD active site contains Walker A and B motifs that are usually found in the P-loop-type ATP/GTP-binding proteins, but not in protein kinases [1, 2, 4]. However, the Walker motifs of the latter differ from the canonical sequences found in other P-loop nucleotide-binding folds [4, 5]. Indeed, in the Walker A motif that is located in the N terminus of BY-kinases, only the GK[S/T] amino acids of the canonical [G/A]X(4)GK[S/T] Walker A motif are well conserved. GS[S/T] amino acids are followed by an additional DXDXR (Walker A’) motif, and then a Walker B motif (consensus sequence hhhhD), which is extended to a [ILVFM](3)DX(2)P sequence [5]. In the C-terminal tail, BY-kinases possess a tyrosine-rich region called the YC-cluster [1–3]. It varies in length (10 to 20 amino acids) and contains several tyrosine residues that correspond to the BY-kinase-autophosphorylation sites [3, 4]. The presence of these four motifs (Walker A, Walker A’, Walker B, and YC) is a typical signature of BY-kinases [4]. BY-kinases of Proteobacteria are also characterized by the existence of a short region rich in Arg and Lys residues, called the “RK cluster”, in the N-terminal part of their cytoplasmic domain [5].

The importance of BY-kinases in the physiology of bacterial cells has been demonstrated in a number of studies. Their best-characterized role concerns the control of extracellular polysaccharide synthesis [2]. Indeed, BY-kinases are involved as co-polymerases in the biosynthesis of capsular and extracellular polysaccharides, which are recognized as important virulence factors in bacteria [2, 6]. In *E. coli*, replacement of the BY-kinase, Wzc, by a mutant form lacking autophosphorylation potential, abolished capsule assembly [7]. It is believed that autophosphorylation/dephosphorylation of BY-kinases is required for proper synthesis and export of polysaccharide polymers [4], which explains the inability of the mutant Wzc to exert its role in capsule formation. Additionally, BY-kinases were found to affect virulence or resistance to cationic antimicrobial peptides, properties that are both associated with capsular polysaccharide synthesis [3]. An example of this is the Etk-mediated phosphorylation of UDP-Glucose Dehydrogenase in *E. coli*, which has been shown to induce resistance to such antibiotics as polymyxin and cationic peptides [4]. Thus, BY-kinases are seen as potential therapeutic targets for combating bacterial pathogens [2]. BY-kinases are not only related to polysaccharide biosynthesis, they are also involved in lysogenization, heat shock response, DNA replication, cell cycle, etc. [3]. Results from Shi et al. [3] confirmed that BY-kinases have evolved a relaxed substrate specificity that allows them to recognize a wide range of substrates with totally different sequences and structure s, and evolve rapidly to adopt new substrates.

Since BY-kinases are not homologs of eukaryotic enzymes and are particularly interesting as therapeutic targets, a specialized bacterial protein tyrosine-kinase database (BYKdb) was developed to store BY-kinase sequences, applying standardized annotations [2]. Despite the importance of BY-kinases, numerous aspects of their biological significance remain obscure. Moreover, the tyrosine-kinase activity of Walker P-loop proteins is assumed to be hardly predictable *in silico*, due to the differences among BY-kinases originating from different bacterial phyla [5].

In the present work, we propose a novel SCMBYK method for predicting and analyzing BY-kinases based on their primary sequences. To our knowledge, SCMBYK is the first open source machine learning tool for BY-kinase classification and characterization. We believe that our tool can significantly increase the rate of amassing knowledge about BY-kinases. SCMBYK uses a newly developed scoring card method (SCM) [8–10] to compute propensities of amino acids and dipeptides in order to discriminate BY-kinases from non-BY-kinases. A dataset consisting of 558 BY-kinases and 544 non-BY-kinases was established to design the SCMBYK predictor. The dipeptide propensity scores were calculated from the differences between the dipeptide compositions of BY-kinases and non-BY-kinases using a straightforward statistical approach. These propensity scores were further optimized using an intelligent genetics algorithm (IGA) [11]. Amino acid propensity scores, obtained from dipeptide propensity scores, were utilized to discover informative physicochemical properties (PCPs) of BY-kinases by exploring the amino acid indices stored in the AA index database [12]. To investigate alternative prediction methods, several typical predictors, such as SVM, decision tree J48, and Naïve Bayes were also implemented. The results from BLAST alignment were compared with machine learning tools. Additionally, 26 models based on SCM were built to predict specific bacterial phyla of target sequences.

The SCMBYK-derived propensity scores of 20 amino acids were further analyzed to identify informative physicochemical properties of BY-kinases, such as: 1) BY-kinases prefer to be composed of α-helices; 2) the content of extracellular regions of BY-kinases is expected to be dominated by Val, Ile, Phe and Tyr residues; 3) BY-kinases structurally resemble nuclear proteins; 4) different domains have different roles in triggering BY-kinase activity. Additionally, the analysis of potential antibiotics for BY-kinases-targeting suggested that Azathioprine (AZA), which is administered to transplant patients, may be able to suppress the virulence of *M. tuberculosis.* Therefore, AZA could be considered as a potential antibiotic for tuberculosis treatment.

## Methods

**Fig. 1:**
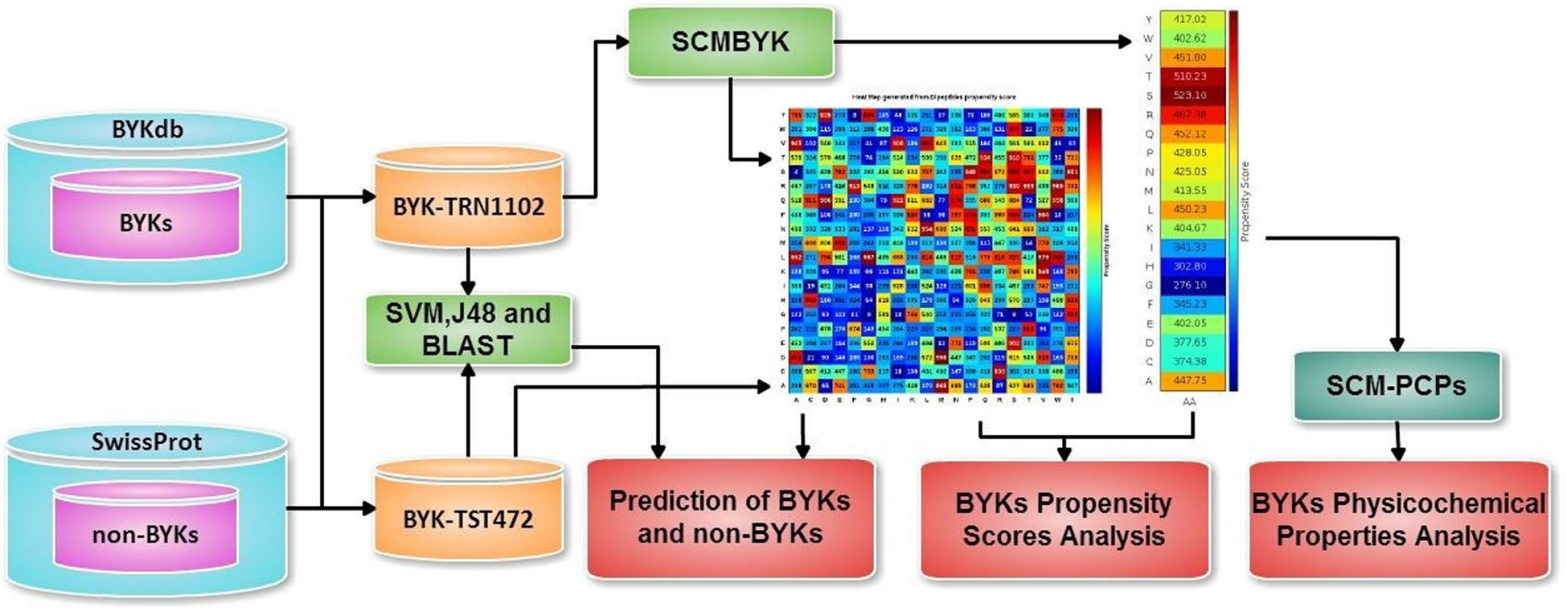
Flowchart of the system design for the prediction and analysis of BY-kinases. BYKs denote BY-kinases, non-BYKs stand for non-BY-kinases

In this work, we propose a novel SCMBYK method, which is a SCM-based predictor and a first analytic tool for the characterization of bacterial tyrosine-kinases. The method relies on a newly established dataset of manually selected BY-kinases from 26 different bacterial phyla and utilizes the SCM algorithm to obtain propensity scores of 400 dipeptides and 20 amino acids. SCMBYK includes SCM-PCP mining method to rank various physico-chemical and biochemical properties for their relatedness to a family of BY-kinases. The method enables visualization of available enzyme structures using the SCM-derived propensity scores and can be applied to predict potential drugs to putative BY-kinases. Figure 1 presents a flowchart of the experimental design, including datasets, methods, and analysis.

### Datasets

The BYK-1580 dataset was compiled from two sources: BYKdb and Swiss-Prot. After reducing sequence identity to < 25%, we created two datasets: BYK-TRN1102 to be used for training the classifier to discriminate between BY- and non-BY-kinases, and an independent test set BYK-TST478, for the evaluation of SCMBYK performance. Table 1 provides the details on both datasets.

**Table 1:** Summary of the training and test datasets

Dataset	BYKP	Non-BYKP	Total
BYK-TRN1102	558	544	1102
BYK-TST472	239	239	478

Here we briefly describe the steps in BYK-1580 dataset creation:

- Step 1: Collect 6,702 BY-kinases of 28 different phyla from BYKdb.
- Step 2: Collect 330,400 non-BY-kinases from Swiss-Prot using the same 28 phyla.
- Step 3: Reduce sequences identity that no pair has more than 25% identity. In this step, two phyla, *Chlamydiae* and *Cloacimonetes*, were excluded because their members can be expressed using corresponding centroids after reducing identity. As a result, 26 phyla were left.
- Step 4: Select 797 BY-kinases to serve as positives.
- Step 5: Select non-BY-kinases to serve as negatives, with the number of negatives from each phylum being the same as the number of positives collected from the same phylum (after Step 3). After performing random pairing of the negative data to positive data according to their phylum, 783 sequences were selected from non-BY-kinases.
- Step 6: The BYK-1580 dataset containing 797 BY-kinases and 783 non-BY-kinases from 26 different phyla was randomly separated into the training (BYK-TRN1102), and test (BYK-TST478) datasets. The details of BYK-TRN1102 and BYK-TST478 are provided in Table 1.

### SCM-based BY-kinase classifier (SCMBYK)

The original SCM algorithm was first proposed by Huang et al. [10] and was consequently applied to discriminate and analyze proteins with various functions [8–10, 13, 14] based on their sequence information. To train the classifier, two FASTA files are expected as the input: one for the positive training data and one for the negative training data. The output is the scorecard file, containing optimized scores of 400 dipeptides. The SCMBYK implementation corresponds to the original SCM algorithm without any major adjustments. The method consists of the following four main phases:

Phase 1: Building of a training set and an independent test set.

Training BYK-TRN1102 dataset was used to optimize the initial matrix of dipeptide propensity scores (DPS) and to determine suitable threshold value for classification of the query sequence as a BY-kinase or non-BY-kinase. Independent BYK-TST478 test set was used to evaluate the prediction model.

Phase 2: Calculation of the initial matrix of dipeptide propensity scores (DPS).

Denote by matrix *DPS*_*(ij)*_ = {*n*_*ij*_} distribution of the amino acid *i* (1 ≤ *i* ≤ 20) followed by the amino acid *j* (1 ≤ *j* ≤ 20). We consider C ∈ {0,1}, representing non-BY-kinases and BY-kinases for binary classification in this article. Initial *DPS* is computed, as follows:

- Step 1: Compute matrices *P*_*(ij)*_ = (*n*_*ij*_|C = 1) and *N*_*(ij)*_ = (*n*_*ij*_|C = 0) of numbers of 400 dipeptides in BY-kinases and non-BY-kinases. For example, *n*_*11*_ (AA dipeptide) is found 2957 times in BY-kinases and 1654 times in non-BY-kinases.
- Step 2: Normalize compositions of dipeptides in matrices *P*_*(ij)*_ and *N*_*(ij)*_ by dividing them by total numbers of dipeptides in each class, as follows:
  1 $$ P(ij)=\left(\left.\frac{n_{ij}}{L_{p-1}}\right|\mathrm{C}=1\right),1\le i,j\le 20 $$
  2 $$ N(ij)=\left(\left.\frac{n_{ij}}{L_{n-1}}\right|\mathrm{C}=0\right),1\le i,j\le 20 $$
  where *L*_*p*_ and *L*_*n*_ represent total dipeptide numbers in BY-kinases and non-BY-kinases, respectively. For example, total number of dipeptides in BY-kinases and non-BY-kinases are 307,246 and 165,921, respectively. Thus, compositions of *n*_*11*_ dipeptide are 0.00962 in BY-kinases and 0.00997 in non-BY-kinases.
- Step 3: Compute initial *DPS* of 400 dipeptide compositions by subtracting each dipeptide score of the non-BY-kinases from the corresponding score of the BY-kinases, as *DPS*_*(ij)*_ = *P*_*(ij)*_ - *N*_*(ij)*_. For example, the score of *n*_*11*_ dipeptide would be −0.00035 (=0.00962–0.00997).
- Step 4: Normalize all scores of the initial *DPS*_*(ij)*_ into the range of [0, 1000]. The score of *n*_*11*_ dipeptide is 296.

The propensity scores for each of 20 amino acids are then computed by averaging the scores of all dipeptides containing these amino acids (ex. for amino acid A average all AX and XA dipeptides, where X – any amino acid).

Phase 3: Optimization of the initial *DPS* using IGA

An intelligent genetic algorithm, IGA [11], is used to optimize initial *DPS* in order to maximize the prediction accuracy and conserve the original sequence information. IGA computes a fitness function, where the area under the ROC curve (AUC) [15], and the Pearson’s correlation coefficient (R-value) between the initial and the optimized propensity scores of 20 amino acids are linearly combined. The weights for the AUC and R value were set based on previous studies [8–10]. (See Eq. 3).

3 $$ \mathrm{Max}.\mathrm{Fit}(DPS)=0.9\times AUC+0.1\times R $$

Phase 4: Prediction of BY-kinases.

The optimal score separating cases from controls in the training dataset is used to set a threshold value of a classifier. When a query protein sequence *P* is encountered in a future, the class prediction is determined by a scoring function, as follows:

4 $$ \begin{array}{ll}S(P)=\hfill & \left\{\begin{array}{l}1, if{\displaystyle {\sum}_1^{400}{w}_i{S}_i}> threshold\hfill \\ {}0, if{\displaystyle {\sum}_1^{400}{w}_i{S}_i< threshold}\hfill \end{array}\right.\hfill \end{array} $$

where *w*_*i*_ and *S*_*i*_ are, the composition and propensity score of dipeptide *i* (1 ≤ *i* ≤ 400), respectively.

SCMBYK used the 10-fold cross validation scheme to obtain optimal propensity scores to differentiate between BY-kinases and non-BY-kinases. The independent test set (BYK-TST478) was employed for evaluation of SCMBYK performance to compare with other classifiers.

### IGA algorithm

The IGA algorithm of the SCM for optimization of the initial *DPS* consists of the following steps:

- Step 1: (Initialization) For initialization, generate randomly *N*_*pop*_ individuals including the initial *DPS*. In this study, *N*_*pop*_ = 40.
- Step 2: (Evaluation) Compute fitness values for all *N*_*pop*_ individuals and determine *Ibest* individual in the population.
- Step 3: (Selection) Select *Ps · N*_*pop*_ individuals to establish a mating pool, using a rank-based selection. In this study, *Ps* = 1.0.
- Step 4: (Crossover) By performing the intelligence crossover operation [15] between *Ibest* and each other individual, determine the best two individuals among two parents and two children as the new children.
- Step 5: (Mutation) Randomly mutate individuals (except *Ibest*) with a mutation probability *Pm* (=0.01), using a real-valued mutation operator.
- Step 6: (Termination) Stop the algorithm if the termination condition is reached, otherwise, go to the Step 2. In this study, 20 generations are used as the stop condition.

### Generic-BYK classifiers

SCMBYK performance regarding identification of BY-kinases was compared with that of three other classifiers, SVM, the J48 decision tree, and Naïve Bayes. The predictors utilized features commonly used in protein function predictions, namely amino acid composition (AAC), dipeptide composition (DPC), and the 531 PCPs from the AA-index database. A 10-fold cross-validation (10-CV) scheme was employed to evaluate the results of all classifiers.

SVM is a golden standard for predicting protein functions, being widely applied in the bioinformatics field. We used LIBSVM (library for support vector machines) [16] to create SVM classifiers with radial basis kernel. The optimal SVM parameters were chosen via a grid search according to the 10-CV accuracy of the training dataset, BYK-TRN1102. The other classifiers were implemented using WEKA package [17], and the default WEKA parameter settings, when applying both the decision tree (J48) and the Naïve Bayes classifiers.

### BY-kinases characterization

BY-kinases were analyzed using the SCM-PCP, as well as propensity score visualization methods. SCM-PCP is a PCP mining method used to identify the important physicochemical properties (PCPs) based on the propensity scores of 20 amino acids [13]. To find a set of PCPs possibly correlated with a considered protein function, we examined the 544 indices representing different PCPs available from the AA-index database. After removing the PCPs containing the value “NA”, 531 PCP indices remained and were considered in this study.

The visualizing method aimed to express the BY-kinase propensity scores to determine their characteristics. The structure coordination files of the proteins were colored according to the amino acid or dipeptide scores, and expressed using PyMOL [18].

### Predicting putative BYKs and their potential drugs

The current study proposes a novel drug repurposing method. The disease-related protein targets are selected using protein function predictors, and then the DrugBank drug database is used to select the potential candidates from a list of approved drugs. In this study, putative BY-kinases in Swiss-Prot were identified by SCMBYK, and then the drugs that potentially interact with the putative BY-kinases were selected using BLASTp. Putative BY-kinases are defined as the sequences that had no detectable transcripts (PE levels from 3 to 5) [19] in Swiss-Prot and are predicted as BY-Kinases using SCMBYK. Then, BLASTp was used to select all the drugs in the DrugBank database that potentially interact with the putative BY-kinases. The detailed procedure consists of the following steps:

- Step1: Retrieve the protein sequences from the 26 phyla hosts from Swiss-Prot.
- Step2: Select the putative BY-kinases that are predicted as BY-kinases by SCMBYK and have PE levels from 3 to 5.
- Step3: Align putative BY-kinases using BLASTp against target sequences listed in DrugBank that are known to interact with approved drugs. The BLASTp uses an E-value threshold of 0.01, while other parameters were set to default [20].

## Results

### Performance comparison of different BYK predictors

BYK-TRN1102 and BYK-TST478 datasets were used to design various BY-kinase classifiers based on different feature types. The proposed SCMBYK method was compared with BLASTp [21], SVM, decision tree (J48), and Naïve Bayes. They utilized amino-acid composition (AAC), dipeptide composition (DPC) and the 531 PCPs from the AA index as features.

To evaluate BLASTp as a BY-kinase predictor, the training dataset was used to build a sequence database. Afterwards, the sequences from the test dataset were treated as query sequences and aligned against the database. The *E*-values ranged from 0.1 to 0.00001. The results are summarized in Table 2, and indicate that the BLASTp method, which is a homology-based tool for predicting protein functions based on their sequence similarities, does not provide satisfying results in predicting BY-kinases. The highest accuracy of 73% was obtained with the E-value cut-off set to 0.1. E-values of 0.01 and 0.001 yielded a lower accuracy (71%). Table 3 lists the prediction accuracies of SVM, J48 decision tree, and Naïve Bayes classifiers with various features. SVM outperformed the other predictors. The highest training accuracy of 97.27% was obtained by the SVM-DPC classifier, while the corresponding test accuracy was 95.76%. The J48 decision tree performed slightly better than the Naïve Bayes method, with the highest training accuracy of 88.75% observed in the J48 /AA-index model. The corresponding test accuracy was 88.35%. The Naïve Bayes predictor produced its best results (84.85% for training accuracy and 86.23% for test accuracy) when it utilized dipeptide composition as a feature set.

**Table 2:** Performance of established datasets as compared for various E-value cut-offs by BLASTp

*E* value	Hit rate	ACC
0.1	74%	73%
0.01	72%	71%
0.001	71%	71%
0.0001	70%	69%
0.00001	69%	68%

**Table 3:** Comparison of the prediction accuracies (%) of BY-kinase predictors

Classifier	Training accuracy	Test accuracy	Specificity	Sensitivity
SVM/DPC	97.27%	95.76%	95.28%	96.23%
SVM/AAC	96.07%	95.13%	96.57%	93.72%
SVM/AA-index	94.56%	94.07%	94.85%	93.31%
J48/DPC	80.94%	82.63%	83.70%	81.50%
J48/AAC	86.48%	89.62%	87.00%	92.30%
J48/AA-index	88.75%	88.35%	90.40%	86.30%
NB/DPC	84.85%	86.23%	86.20%	86.30%
NB/AAC	77.22%	78.18%	67.80%	88.80%
NB/AA-index	76.50%	71.19%	90.00%	51.90%
SCMBYK	97.55%	96.73%	98.00%	96.00%

Table 4 presents the results from 10 independent runs of the SCMBYK method on the BYK-TRN1102 and BYK-TST478 datasets. The scoring card used to build SCMBYK predictor was chosen as the one having the fitness score closest to the average fitness score. Hence, Experiment #10 with a training accuracy of 97.55% was chosen as a model for SCMBYK. The SCMBYK method achieved a test accuracy of 96.73%, a Matthews Correlation Coefficient (MCC) of 0.93, a sensitivity of 0.96, and a specificity of 0.98. Using IGA algorithm improved training and test accuracies of the initial scoring card from 87.18 to 97.55% and from 81.57 to 96.73%, respectively. The corresponding threshold value was raised from 406 to 468. The histogram in the Additional file 1 shows that the BY-kinases and non-BY-kinases sequence’ scores in a test dataset (BYK-TST478) became more separable after the optimization by IGA.

**Table 4:** The performance of 10 independent runs using BYK-TRN1102

	Fitness	Training ACC (%)	Test ACC (%)	MCC	Sen.	Spe.	Threshold
#1	99.21	97.36	96.27	0.93	0.97	0.95	474
**#2**	**99.24**	**97.55**	**96.55**	**0.93**	**0.97**	**0.96**	**475**
#3	99.21	97.82	96.36	0.93	0.97	0.95	486
#4	99.08	97.73	96.82	0.94	0.98	0.95	485
#5	99.32	97.64	96.82	0.94	0.99	0.95	484
#6	99.16	97.36	96.00	0.92	0.99	0.93	460
#7	99.02	97.00	96.27	0.93	0.95	0.98	496
#8	98.94	97.18	96.18	0.92	0.98	0.95	470
#9	99.08	97.82	96.91	0.94	0.97	0.97	464
**#10**	**99.20**	**97.55**	**96.73**	**0.93**	**0.96**	**0.98**	**468**
AVEG	99.19	97.50	96.49	0.93	0.97	0.96	476.20

The bold indicate the performances of SCMBYK

Our results suggest, that SCMBYK method outperformed other classifiers, including SVM-DPC in terms of both accuracy, sensitivity and specificity. High prediction performance of SCMBYK can be explained by the fact, that dipeptide composition is an optimal and representative feature for the task of discrimination between BY-kinases and non BY-kinases. This also follows from the high training accuracies of SVM-DPC, J48-DPC and NB-DPC classifiers, being 97.27, 80.94 and 84.85% respectively.

Furthermore, the SCM-based SCMBYK method have the following advantages over other classifiers: (i) Distinctive to SVM, which is a prevalent method for protein classification, SCM does not function like a black box. The biological interpretation of the model is more straightforward, as long as it generates propensity scores of dipeptides, which can be further analyzed. (ii) Amino acid propensities, derived from SCM allow to rank physico-chemical properties relevant to a given protein family and inspire biological application. (iii) In terms of prediction accuracy, the SCM method is comparable with SVM.

### SCMBYK performance for identifying BY-kinases using different phyla of datasets

The leave-one-phylum-out test is applied to evaluate the ability of SCMBYK to predict BY-kinases from novel phyla, i.e., from phyla that were not included in the training dataset. For each of the 26 phyla included in the BYK-1574, training was based on a dataset composed of the BYK-1574 sequences minus the sequences corresponding to the specific phylum, the latter forming the independent test dataset for the particular phylum. According to the results (Additional file 2), the mean training accuracy and test accuracies achieved were 97.00 and 97.39%, respectively. The MCC, sensitivity and specificity of test were rather high. Therefore, we conclude that SCMBYK performs well at distinguishing between BY-kinases and non-BY-kinases of novel phyla.

### Analysis of SCMBYK-derived propensity scores

The SCMBYK predictor operates by calculating dipeptide (DP) and amino acid [22] propensity scores of BY-kinases and non-BY-kinases. Calculated propensities quantitatively represent the impact of each dipeptide and amino acid on the structure and functionality of a given protein class. We used visualization techniques to color structures of known BY-kinases according to SCMBYK-derived DP and AA scores.

### Dipeptide propensity scores analysis

**Fig. 2:**
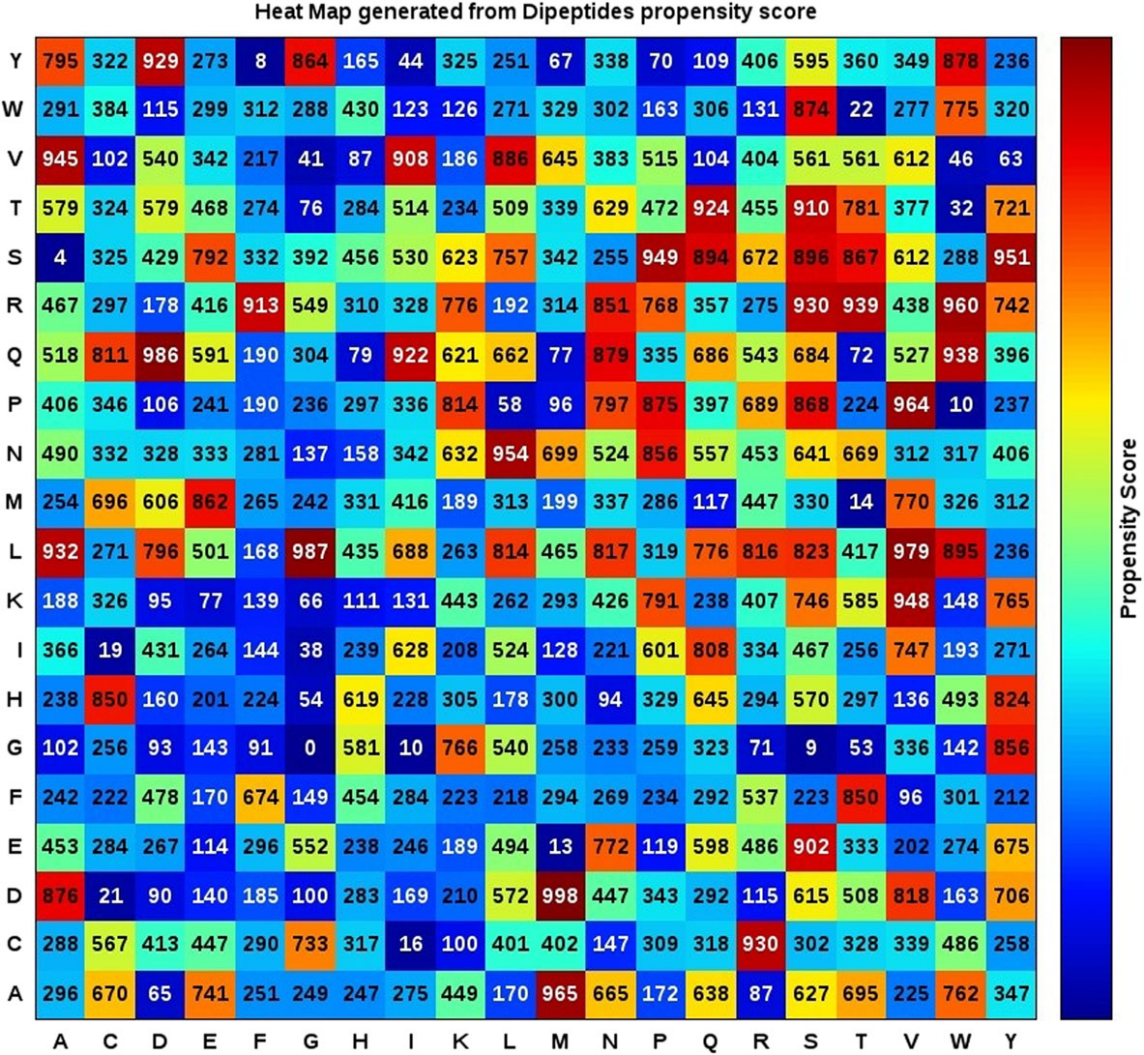
Heat map of the SCMBYK propensity scores of dipeptides

Figure 2 shows a heat map of the SCMBYK-derived propensity scores of 400 dipeptides as BY-kinases and non-BY-kinases. The five top-ranked dipeptides are DM, LG, QD, LV, and AM, with respective scores of 998, 987, 986, 979, and 965. The five dipeptides with the lowest scores are GG, SA, YF, GS, and GI, scored 0, 4, 8, 9, and 10, respectively.

**Fig. 3:**
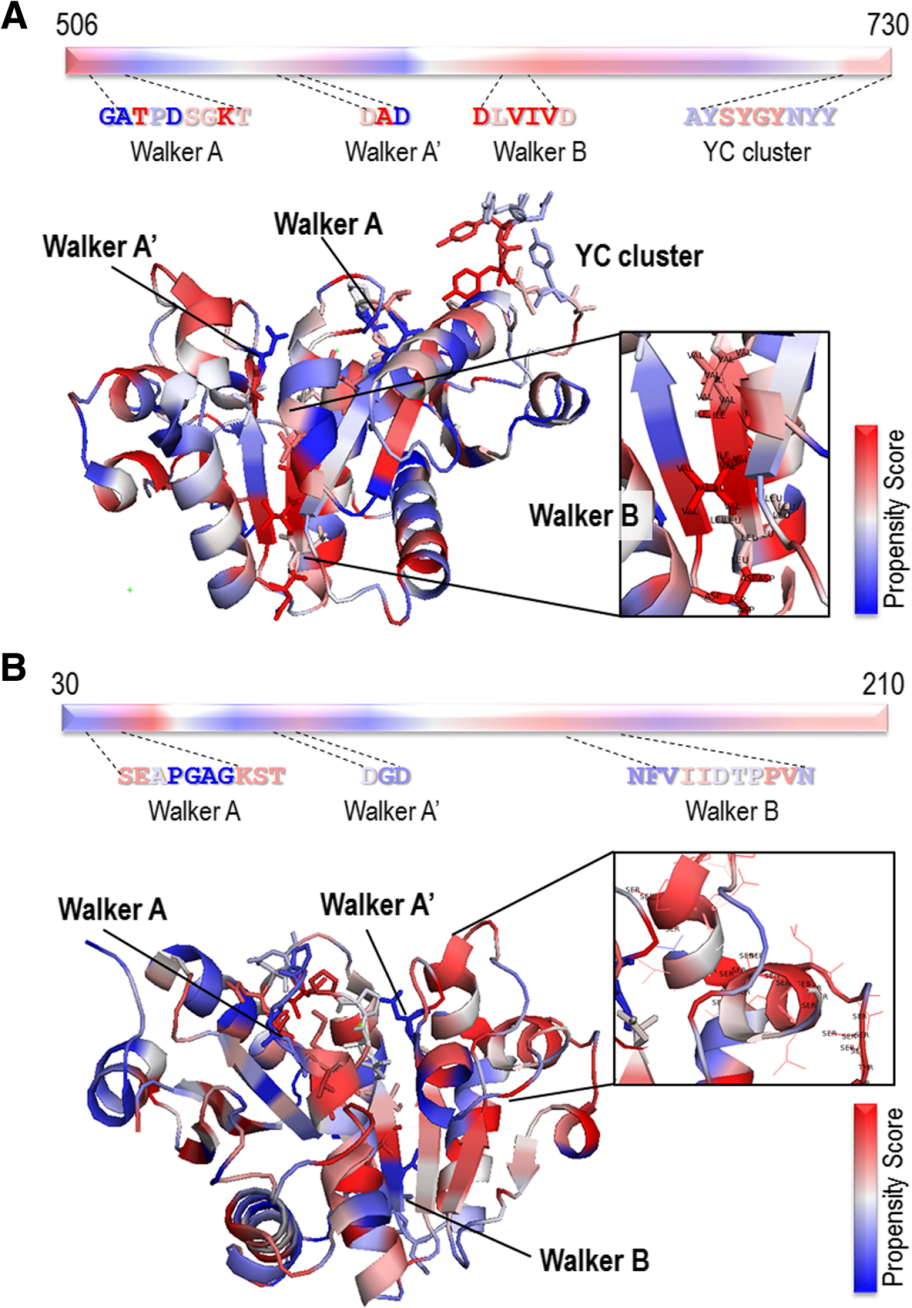
The DP visualization of BY-kinase structures. **a** Visualization of the overall structure of the Etk kinase domain (PDB code 3CIO), and a close view of the high-score Walker B motif. **b** CapB2 DP visualization (PDB code 3BFV), and a close view of the highly scored stretch between the Walker A’ and Walker B motifs. The *red* color is used to mark the positions of high-score dipeptides, in contrast to the low-score dipeptides, which are colored *blue*

Figure 3 presents the visualization of the distributions of DP propensity scores of the cytoplasmic domains of Etk [23] in the gram-negative bacterium *E. coli*, and of CapB2 [24], which is the cytoplasmic, catalytically active BY-kinase-subunit in the gram-positive bacterium *S. aureus*.

Visualization of the Etk and CapB2 structures according to the DP scores shows that both dark blue and deep red regions are present and spread equally on α-helices and β-strands. Among the signature motifs of the Etk kinase, the Walker B motif appears to be composed of the most highly-scored DPs (Fig. 3a). On the other hand, no motif from the CapB2 active site was colored completely in red. However, a long stretch of residues between the Walker A’ and Walker B motifs, starting from Ser95 / Ser96 and spreading up to Ser134, display DP with a minimum blue hue (Fig. 3b). Clearly, regions colored red contribute more to the SCMBYK scores, which determine whether the overall sequence will be predicted as a BY-kinase, compared to the blue ones.

### Amino acid propensity scores analysis

The amino acid scores of BY-kinases were calculated from dipeptide propensities using a straightforward statistical approach. These scores reflect the frequency of each amino acid in a polypeptide chain as well as its unique impact on the functionality of a protein. However, it is not a trivial task to delineate these correlations, as BY-kinase sequences display a high level of substitution saturation which allows them to maintain their status as platforms for adopting new substrates [3].

Additional file 3 presents the 20 amino acid propensities as well as the AA compositions of BY-kinases and non-BY-kinases. The high correlation coefficient (*R* = 0.99) between the propensity scores of amino acids and composition difference in BY-kinases and non-BY-kinases indicates that SCMBYK-derived AA propensities are effective for discriminating between BY-kinases and non-BY-kinases.

The five amino acids with the highest SCMBYK scores include Ser, Leu, Gln, Arg, and Thr with scores of 594, 571, 522, 500, and 475 respectively. The five amino acids with the lowest SCMBYK scores are Gly, Phe, His, Lys, and Trp, with scores of 287, 305, 342, 371, and 373 respectively. Remarkably, all high-score amino acids are polar, with the exception of Leu. Furthermore, most aromatic residues obtained low scores, with only Tyr being in the middle of the range.

Recently, much effort has been put to solve crystal structures of BY-kinases. Analysis of crystallized cytoplasmic domains of the *E. coli* tyrosine kinase Etk and of its orthologue CapB (which is the endoplasmic, tyrosine-kinase active subunit of the BY-kinase) from the Firmicute *S. aureus*, gave interesting clues regarding the role of several amino acid residues in the active sites of BY-kinases [4, 25]. The conserved Lys and Thr residues of the Walker A motif, the two conserved Asp residues of the Walker A’ motif, and the single conserved Asp of the B motif, interact with the phosphate moiety of the bound nucleotide and the associated magnesium ion [4]. Replacing a P-loop Lysine with Methionine is known to inhibit the phosphate-transfer activity of the shikimate kinase without impairing ATP binding [26]. The side chain of the penultimate Phe221 residue of CapA, which is stacked on the base part of the bound ADP molecule, associates with it through a strong hydrophobic interaction, stabilizing nucleotide binding and explaining the activation mode of CapB [4]. The study performed on the Wzc BY-kinas of *E. coli* showed that phosphorylation of the Tyr569 residue results in an increased protein-kinase activity, and can in turn phosphorylate YC [5]. Additionally, the second Asp of the Walker A’ motif (hhhhDXDXR) directly interacts with the phosphorylatable hydroxyl of the Tyr, most probably acting as an acid catalyst [4]. The high-resolution crystal structure of the non-phosphorylated form of CapB2 showed that CapB2 forms a ring-shaped octamer [5]. The conserved Arg of the Walker A’ motif plays a crucial role in stabilizing the octamer [4]. Additionally, Asp77 and Asp79 of the Walker A’ motif, as well as Asp157 and Pro159 of the Walker B motif, are conserved in this protein [5].

In the SCMBYK scale both Arg and Thr are among the five highest-scoring residues. They are followed by the middle-score residues, Asp, Tyr and Pro. Although Lys and Phe were mentioned previously as functionally crucial in BY-kinase active sites, they are low-score residues according to the SCM method.

The propensities obtained from the SCMBYK predictor can be efficiently utilized for mutagenesis studies. Since their role of in bacterial extracellular polysaccharide synthesis makes them potential therapeutic targets,, mutations that can block these enzymes can affect bacterial virulence.

**Fig. 4:**
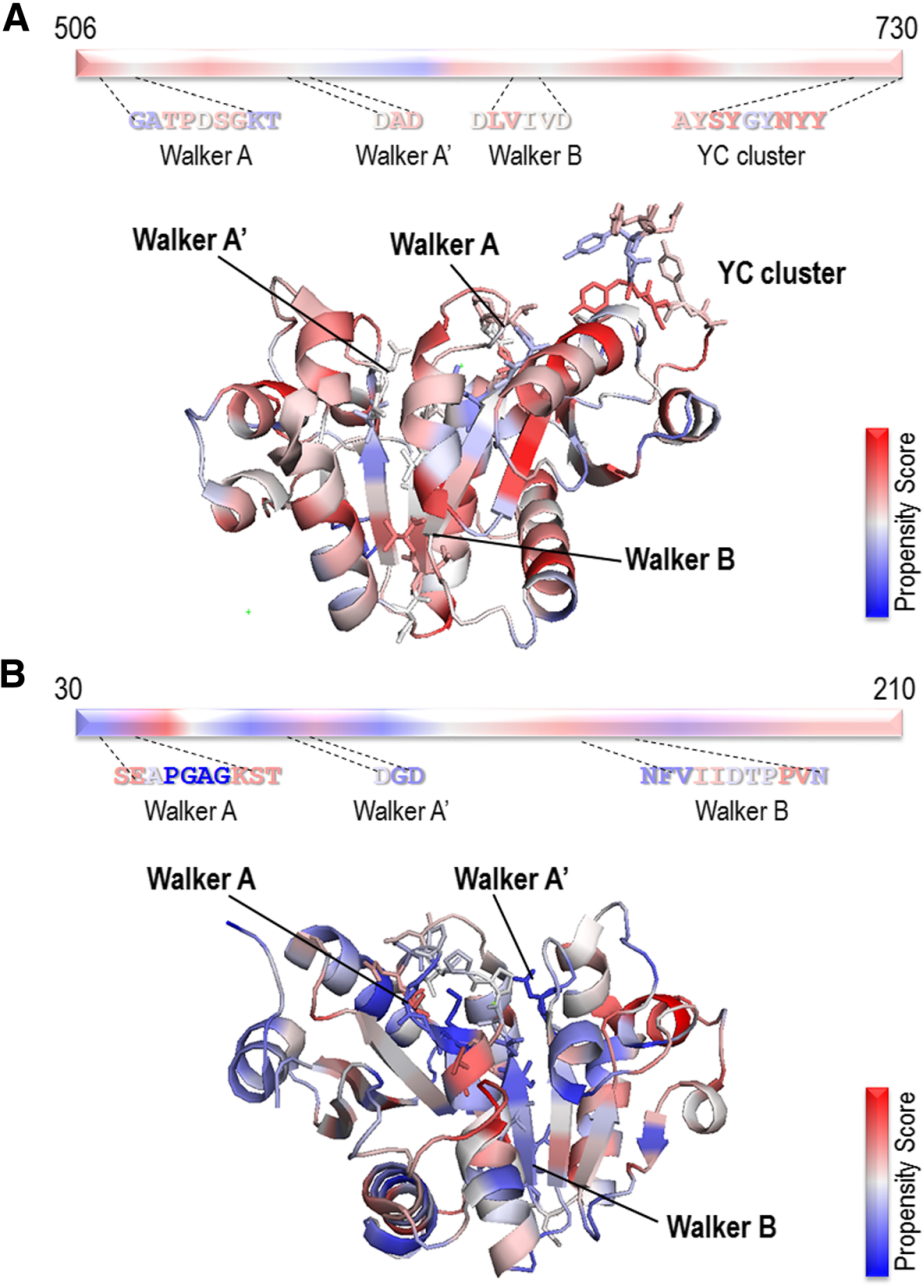
The AA visualization of BY-kinase structures. **a** AA visualization of the overall structure of the Etk kinase domain (PDB code 3CIO). **b** CapB2 AA visualization (PDB code 3BFV). *Red* color represents the positions of highly-scored amino acids, in contrast to the low-scored AA, which are colored in *blue*

For the visualization of the distributions of AA propensity scores, the catalytic, intracellular domains of Etk in *E. coli* and CapB2 in *S. aureus* were chosen. In Fig. 4, the overall tones of the Etk kinase structure visualized according to the SCMBYK-derived AA scores are more homogenous without clear extremes either in high- or low-scores sides. Here, Walker A motif from the active site contained the most low-scored residues (Fig. 4A). The tones of the CapB2 structure, however, are more blue than red. All signature motifs contained predominantly low-scored residues (Fig. 4B).

### SCM scores of BY-kinases’ motifs

As mentioned previously, catalytic, intracellular domains of BY-kinases, which contain Walker A, A’ and B motifs are required for their kinase activity. Therefore, we used annotated domains from Pfam database to calculate corresponding SCMBYK scores of BY-kinase active fragments. The SCMBYK scores were determined in a positive subset for the PF01656 Pfam domain, which included all signature motifs. According to our results, the average PF01656 motif scores were 508, and the average scores of the whole sequences were 495. Both values are higher than the model threshold of 468. The difference between the average PF01656 motif scores and whole sequence scores proved to be significant (*p* < 0.05). Hence, the fragments with signature motifs play a crucial role for the identification of BY-kinases by our model.

**Fig. 5:**
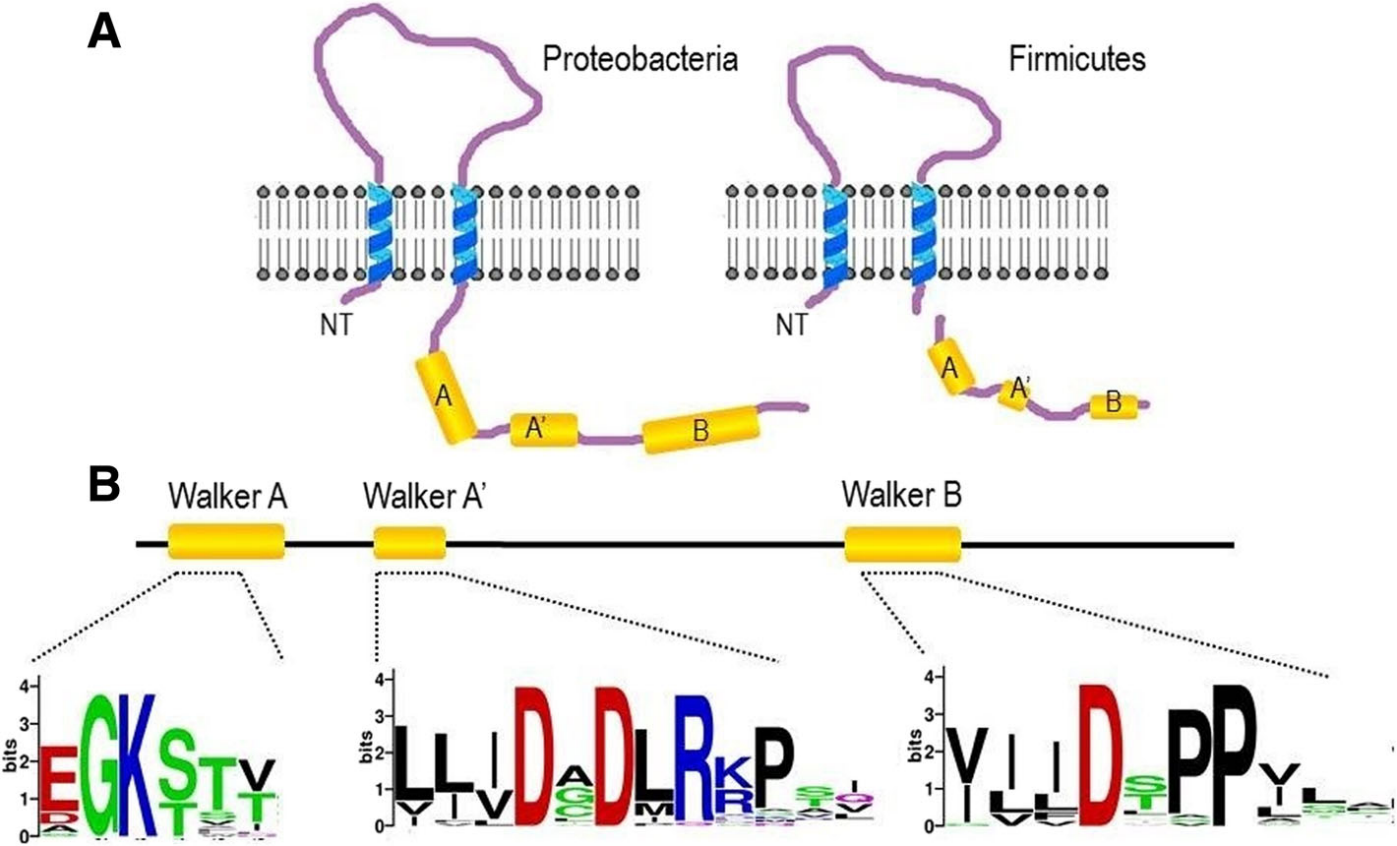
Schematic organization of BY-kinases and their active sites. **a** Organization of BY-kinases in Proteobacteria and Firmicutes. Walker motifs (A, A’ and B), extracellular hairpin domains, and transmembrane spans are colored yellow, purple, and blue, respectively. In Proteobacteria, the extracellular loop and the intracellular domain are parts of the same protein, whereas in Firmicutes they are linked via specific protein-protein interactions. **b** Sequence logos of signature amino-acid sites of top-30-scored BY-kinases. “GK” in Walker A, “DXDXR” in Walker A’, and “DXPPX” in Walker B are indicated by larger letters

Furthermore, we estimated the average SCMBYK-derived scores for the signature motifs of the top-30 scored sequences that are selected depending on the score of each sequence in training dataset. The average scores were 668, 572, and 603 for Walker A, A’, and B, respectively. This is much higher than the model threshold of 468. Thus, amino acid consensus sites in BY-kinases play crucial role for the BY-kinase prediction. Sequence logos of signature motifs of top-30 SCMBYK-scored proteins are presented in Fig. 5. Sequence logos were generated with the Weblogo program [27].

### Overall α-helical preference of BY-kinases

The highest positive correlation (*p* = 0.53) of BY-kinases’ amino acid propensities was obtained with the MAXF760106 scale from the AA index, which represent the Normalized frequency of alpha helices. MAXF760106 contains the indices of 20 amino acids related to the frequency of their being topologically in alpha regions as one of five possible conformational states [28]. Originally, Maxfield and Sheraga [28] used data from 20 proteins with known three-dimensional structures to determine specific backbone dihedral angles for each amino acid residue [28]. Consequently, Kidera recalculated these values using a different set of proteins, and normalized given values by the residue total number.

The residue conformational states defined in terms of the backbone dihedral angles can be further used by prediction algorithms to assign starting conformations of proteins from their amino acid sequences following the energy-minimization method. The positive correlation between SCM-derived scores and the MAXF760106-scale indices could account for the topological preferences of BY-kinases in terms of residue conformational states. In this regard, we can state that alpha regions are the most preferable structures among BY-kinases. Notably, three out of the five SCMBYK top-score residues, Ser, Arg and Thr, are also among the top-5 residues in the MAXF760106 scale. Additionally, Gly which has a low score in the MAXF760106 scale, is also one of the five bottom-5 property is one of the five SCM lowest-scoring amino acids (Gly, Phe, His, Lys, and Trp).

Determination of the structure of the extracellular domain of BY-kinases can provide important clues for its function. Given their modular organization, it is tempting to speculate that BY-kinases topology can be associated with their specific functionality. There are clear parallels between BY-kinases’ overall α-helical preferences and the predicted presence of extended β-structures in the extracellular domains, which implies an additional sensor-activity role for these domains, especially in the BY-kinases of Proteobacteria [4, 6]. However, no high-resolution data on the external domains of BY-kinases are available to date [5]. To estimate the preference of BY-kinases, two sequence-based secondary structure predictors were used, SOPMA [29] and NetSurfP [22]; the results are presented in Additional file 4. According to SOPMA, BY kinases have significantly (*p* < 0.001) higher (45.44) α-helical-structure content compared to non-BY-kinases. NetSurfP also detects a significant (*p* < 0.001) difference between BY- and non-BY-kinases, with the respective a-helical-content values being 48.13 and 42.05.

### Specific BY-kinase topology

The RACS820107 property is defined in the AA index as the “average relative fractional occurrence in A_0_ (i-1)” and refers to structural features of polypeptide backbones related to distributions of the 20 amino acids within the polypeptide chain [25]. More precisely, it defines the role of each amino acid in the formation of A_0_ regions.

Based on the concepts of differential geometry, the protein backbone structure is viewed through a virtual-bond representation, in which the C^α^ atoms of successive residues are considered to be connected by imaginary bonds. The four successive C^α^ atoms are considered to be the smallest length of backbone over which the chain can be folded [30]. At the four-C^α^ length scale, a-helical structures appear nearly flat and called A_0_ regions [25, 30]. Rackovsky et al.[25] presented a scale, which determines the effect of every single amino acid in forming A_0_ structures if these residues are located at the third position of a four-C^α^ unit.

By comparing these values with the SCM-generated amino acid scores, a negative correlation has been obtained (*p* = −0.40). Thus, it can be assumed that BY-kinases do not favor the formation of flat α-helical structures. This assumption, however, is not extended to other types of α-structures (right-handed and left-handed).

Moreover, in an attempt to correlate composition and structure of polypeptide chains, Rackovsky et al. [25] further defined two groups of amino acids which are responsible for the formation of different structures. Group I residues (Ser, Thr, Val, Arg, Gln, Leu, Ala, Asp, Glu, Lys, Met, Ile, and Phe) favor the formation of A structures (A_R_ helices and A_R_, A_L_, and A_0_ bends) in four-C^α^ units, when located at the second position, and E_0_ and A_R_ structures, when located at the third position. Group II residues (Pro, Gly, His, Tyr, Cys, Asn, and Trp) are responsible for the formation of E_0_ and A_R_ structures when located at the third position.

The correlation results between the RACS820107 scale and the SCM-derived scores allow for the conclusion, that group I and group II amino acids may play similar roles in the formation of BY-kinase- structures, such as bends, helices, and extended regions.

### Amino acid composition of BY-kinases extracellular regions

The NAKH920103 property is the AA composition of EXT of single-spanning proteins and provides the average amino acid composition of the extracellular regions of single-spanning transmembrane proteins [31]. The SCM-generated amino acid scores, positively correlated (*p* = 0.50) with the NAKH920103 scale. This scale was derived by the results of Nakashima et al. [31], who studied 73 peptides longer than 50 residues, from 45 single-spanning membrane proteins. The BY-kinases can be divided into two groups based on their architecture. In Proteobacteria, these enzymes are found in the form of membrane proteins with large outside loops linked to the catalytic cytoplasmic domains [1, 4]. in contrast, BY-kinases of Firmicutes possess the cytoplasmic catalytic domain in a polypeptide that interacts with a separate membrane protein, homologous to the extracellular domain of proteobacterial BY-kinases [4].

The positive correlation results suggest that cytoplasmic (CYT) and extracellular (EXT) regions of BY-kinases have different amino acid compositions. More specifically, the extracellular regions are expected to be dominated by residues favoring the β-sheet structure, such as Val, Ile, Phe and Tyr. Interestingly, aromatic residues (Trp, Tyr and Phe) are preferred on the extracellular side of membranes, whereas charged residues, both basic (Arg, Lys) and acidic (Glu, Asp), are preferentially sited on the cytoplasmic side [31]. These results are in accord with previous studies that performed structural predictions and showed that the extracellular domains of BY-kinases from Proteobacteria tend to favor β-structures [6].

### BY-kinases resemble nuclear proteins

The CEDJ970105 property is described in the AA index as the “composition of amino acids in nuclear proteins” [32]. The amino acid indices of CEDJ970105 property were derived from a set of sequences with verified cellular locations, and represent the scores of the frequencies for each amino acid residue to be found in one of five protein-location classes. Prokaryotic proteins that interact with DNA were classified as “nuclear”. The CEDJ970105 indices correlate positively (*p* = 0.48) with the SCM-derived propensity scores for BY-kinases. This is in accord with previous studies showing that the active sites of BY-kinases share signature Walker A and B motifs with a number of ubiquitous ATP/GTPases [1, 2, 4], and one should keep in mind that nucleotides not only serve as the building blocks for the transmission of genetic information, but are also involved in energy transfer and storage. Moreover, nucleotide-binding folds are ancient and widespread [33]. According to Grangeasse et al. [4], BY-kinases exhibit significant sequence similarity with nucleotide-binding motifs of arsenite ATPases (ArsA) and MinD proteins, a fact that leads to the hypothesis that they have all evolved from the same ancestral bacterial ATPase [3].

Notably, both SCM-derived and CEDJ970105 scales rank Ser as a top-score residue. Furthermore, among the SCM five top-score amino acids (Ser, Leu, Gln, Arg, and Thr), there are two, Arg and Leu, that are also among the top five in the CEDJ970105 scale. Additionally, two of the low-score CEDJ970105 residues, His and Trp, are among the five lowest-score SCM amino acids (Gly, Phe, His, Lys, and Trp).

However, our results indicate that BY-kinases could also possess similarities with nucleotide-binding motifs of nuclear proteins. As the whole cluster of BY-kinases has yet no equivalents, even among their close structural homologues, other templates must be sought [4]. The positive correlation leads us to the assumption that a considerable degree of similarity in amino acid composition exists between BY-kinases and the proteins characterized as nuclear in a previous paper [32]. These proteins are generally poor in hydrophobic (especially aromatic) amino acid residues and rich in charged residues. They also have a high content of serine, threonine, proline, asparagine and glutamine residues [32].

### BY-kinases as anchored proteins

The SCM-derived amino acid scores also show a positive correlation (*p* = 0.43) with the CEDJ970102 property, which can be described as the “composition of amino acids in anchored proteins” [32]. Out of the five SCM top-score amino acids (Ser, Leu, Gln, Arg, and Thr), two (Ser and Leu) are also among the top five in the CEDJ970102 scale. Furthermore, two of the low-score CEDJ970105 residues, His and Trp, are also among the five SCM lowest-scoring amino acids (Gly, Phe, His, Lys, and Trp).

BY-kinases possess a transmembrane domain and cannot be considered anchored proteins [1]. However, the positive correlation was obtained with the scale, corresponding to anchored proteins, rather than integral membrane proteins [32]. It should be mentioned that protein kinase phosphorylation events in eukaryotes are tightly regulated by anchoring proteins, as in the case of the complexes consisting of protein kinase A (PKA) and A-kinase anchoring proteins (AKAPs). AKAPs stimulate PKA holoenzymes and bring them in a close proximity with a variety of signaling partners. Additionally, AKAPs are conformationally and compositionally flexible and able to modulate multiple signal pathways [34].

BY-kinases in firmicutes and proteobacteria differ with respect to how the transmembrane domains interact with the catalytic domains. In proteobacteria, the two domains are located in the same polypeptide chain, while in Firmicutes they are linked through a specific interaction of helices [1]. Hence, not all species adhere to the “one-chain” model. As pointed out by Grangeasse et al. [1], the transmembrane protein in firmicutes influences the kinase activity itself, whereas in proteobacteria the situation is less clear. Based on the correlation results and our previous observations on the influence that the BY-kinase TAD domain exerts on enzyme activity, we can assume that there are close parallels between its role in triggering the BY-kinase activity of the CD domain and the function of anchored proteins, especially the anchored proteins that interact with kinases in eukaryotes [1]. Depending on the species, the TAD domain can have different signal input. This, however, needs further experimental verification.

## Discussion

### Predicting potential drugs for BY-kinases

Tyrosine phosphorylation by BY-kinases has been shown to regulate many cellular processes in bacteria, such as virulence and proliferation [35]. Due to the arising predominance of antibiotic-resistance bacteria, BY-kinases are considered as possible targets for curing bacterial infections. The results of Sajid et al. [36] indicate that the host immune systems affect the responses of bacteria, which use signal proteins such as kinases or phosphatases to sense the environment and transduct signals. Discovery of more chemical molecules that can prevent bacteria from modifying their overall behavior in response to the host would be helpful in the fight against antibiotic-resistant bacteria. In the pharmaceutical industry, development of alternative purposes for marketed drugs is not a new strategy. Andronis et al. [37] remarked that the methods mainly used for drug repurposing are based on literature mining and ontologies. In this study, an alternative strategy that uses SCMBYK to select potential BYKs and select approved chemical molecules that may possibly interact with BYKs, is proposed as a novel method of drug repurposing.

The results included 27,474 interactions derived from 5,022 putative BY-kinases and 586 approved drugs (from the DrugBank database) as shown in the Additional file 5. The putative BY-kinases annotated with PE level 5 are listed in Table 5. There are three proteins, O0531, P76123, and Q92HC9, from three different hosts, *E. coli*, *H. influenza*, and *R. conorii*, respectively. O0531is is annotated with a function description of “Truncated acetolactase synthase; no longer catalytically active” (Additional file 5), while the other two, P76123 and Q92HC9, have unknown functions. Beside antibiotics (DB00336 and DB01091), the selected drugs include anti-cancer (DB00336) and antifungal agents (DB00735 and DB00857), drugs for the treatment of hypertension (DB09242) and eye disease (DB03147), as well as a pharmaceutical agent used in spasticity management (DB00697).

**Table 5:** The putative BY-kinases and the potential drugs

Drug ID	Drug name	Target protein	Organism	Score
DB00724	Imiquimod	P76123	*Escherichia coli .*	478.39
DB09242	Moxonidine	P76123	*Escherichia coli .*	478.39
DB00697	Tizanidine	P76123	*Escherichia coli .*	478.39
DB00336	Nitrofural	O05031	*Haemophilus influenzae*	474.70
DB03147	Flavin adenine dinucleotide	O05031	*Haemophilus influenzae*	474.70
DB01091	Butenafine	Q92HC9	*Rickettsia conorii*	472.28
DB00857	Terbinafine	Q92HC9	*Rickettsia conorii*	472.28
DB00735	Naftifine	Q92HC9	*Rickettsia conorii*	472.28

Finding new antibiotics against antibiotic-resistant *Mycobacterium tuberculosis*, the bacterium that causes tuberculosis, is also of extreme importance. The World Health Organization (WHO) estimates that 9.6 million people worldwide suffered from tuberculosis during 2014, and 480,000 of them were infected with multiple-drug-resistant species, which are becoming a major threat to global public health security [38]. Hence, many studies emphasize the importance of finding new antimicrobial drugs [38] or identify new BY-kinases as potential drug targets [4]. Here, we analyzed the putative BY-kinases from *M. tuberculosis*, as shown in the Additional file 5. The putative BY-kinases having a PE level of 3 from *M. tuberculosis* were selected because of the absence of kinases with PE levels 4 and 5. Consequently, 15 putative BY-kinases and 35 drugs were chosen. Among these drugs, some have already been reported to possess anti-tuberculosis properties, such as mercaptopurine. Notably, the results of this study present Azathioprine (AZA), which is used to manage transplant patients, as a drug that may suppress the virulence of *M. tuberculosis*. This could provide an alternative explanation for the observations of Mercadal et al.[39] who reported that patients with a long-lasting renal allograft developed tuberculosis after switching from AZA to mycophenolate, and suggested that mycophenolate was responsible for late reactivation of dormant tuberculosis. Our results, according to which AZA may interact with BY-kinases and suppress the virulence of *M. tuberculosis*, suggest that it was the removal of AZA, and not the introduction of mycophenolate, that led to the appearance of tuberculosis in the patients that switched medication.

## Conclusions

Since their discovery BY-kinases have been receiving a growing amount of attention. This is especially true for the biomedical field, where they are seen as promising targets for anti-bacterial drug design. In this study, several methods, including the homology-based BLASTp, SVM, the J48 decision tree, and Naïve Bayes, were applied to predict BY-kinases based on their sequence information. The efficiency of these classifiers was compared to that of a novel SCMBYK method, which yielded an excellent prediction performance. Furthermore, our PCP mining method revealed a high correlation between the propensity scores of 20 amino acids and such PCPs as: MAXF760106, RACS820107, NAKH920103, CEDJ970105, and CEDJ970102. In summary, 1) BY-kinases prefer to be composed of α-helices; 2) the content of extracellular regions of BY-kinases is expected to be dominated by such residues, as Val, Ile, Phe and Tyr; 3) BY-kinases structurally resemble nuclear proteins; 4) different domains have different roles in triggering BY-kinase activity. Since the BY-kinases are highly correlated to the virulence of bacteria, looking for new drugs would be helpful for the treatment against the antibiotic-resistant bacteria. This study identified three approved drugs that are currently not used as antibiotics. Notably, as azathioprine is predicted to suppress the virulence of *M. tuberculosis* and, it could prove to be a potential antibiotic for tuberculosis treatment*.*

To the best of our knowledge, enzyme-specific SCMBYK classifier is the first open source machine learning tool for the BY-kinase classification and characterization. Compared to earlier SCM-based methods [10, 13, 14], SCMBYK is more strictly formulated for the purpose of characterization of BY-kinases, as long as it relies on a carefully selected dataset of 26 different bacterial phyla. With the advent of next-generation sequencing, the rate at which protein databases grow is very fast. The leave-one-phylum-out experiment has proved, that our algorithm can effectively predict BY-kinases even if their bacterial hosts were not included into the training dataset. Moreover, we show that SCMBYK classifier scored BY-kinase signatures, namely Walker A, A’ and B motifs, much higher than its threshold value, showing that our method can be helpful in identification of meaningful motifs of BY-kinases. Thus, we believe that SCMBYK is a useful tool to guide experimental studies on putative BY-kinases, and is very important for the various applications in medicine and pharmacology.

## Additional files

Additional file 1: Figure S1. The histogram of the BY-kinase and non-BY-kinase propensity scores in the test data. (A) Statistical DPS without optimization. (B) optimized DPS. (PNG 22 kb)
Additional file 2: Table S1. Leave-one-phylum-out test is applied to evaluate SCMBYK. (DOCX 14 kb)
Additional file 3: Table S2. The propensity scores and composition (%) of amino acids in BY-kinases. (DOCX 13 kb)
Additional file 4: Table S3. The averaged a-helices contents. (DOCX 15 kb)
Additional file 5: Showing interactions between 5,022 putative BY-kinases and 586 approved drugs (from the DrugBank database). Open in Excel. (XLSX 1778 kb)
