# Supplementary figures and images for: SCMBYK: prediction and characterization of bacterial tyrosine-kinases based on propensity scores of dipeptides
Source: BMC Bioinformatics. 2016 Dec 22;17(Suppl 19):514. doi: 10.1186/s12859-016-1371-4 (PMC5260027; doi:10.1186/s12859-016-1371-4)

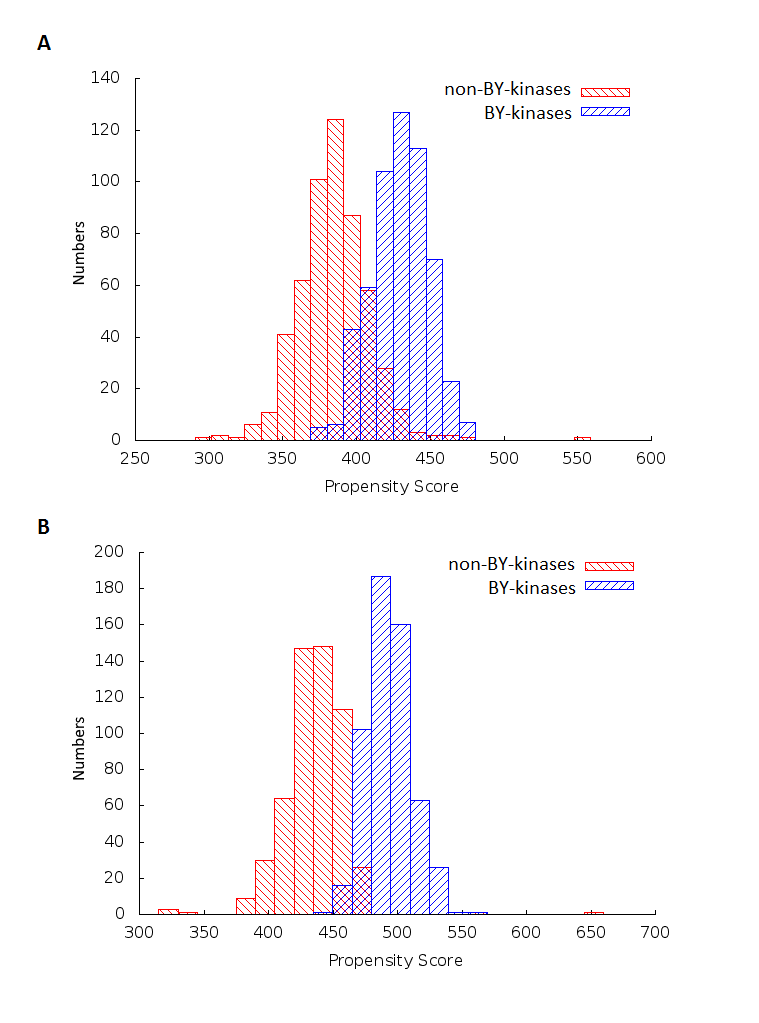

Supplement: Additional file 1: Figure S1. — The histogram of the BY-kinase and non-BY-kinase propensity scores in the test data. (A) Statistical DPS without optimization. (B) optimized DPS. (PNG 22 kb) [file 12859_2016_1371_MOESM1_ESM.png]
