# Supplementary material for: SCMBYK: prediction and characterization of bacterial tyrosine-kinases based on propensity scores of dipeptides
Source: BMC Bioinformatics. 2016 Dec 22;17(Suppl 19):514. doi: 10.1186/s12859-016-1371-4 (PMC5260027; doi:10.1186/s12859-016-1371-4)
Supplement: Additional file 2: Table S1. — Leave-one-phylum-out test is applied to evaluate SCMBYK. (DOCX 14 kb) [file 12859_2016_1371_MOESM2_ESM.docx]

**Table S1 Leave-one-phylum-out test is applied to evaluate SCMBYK.**

| **Fitness Score(%)** | **25 phyla**  **Train Acc.(%)** | **Phylum ID** | **Test Acc.(%)** | **MCC** | **Sensitivity** | **Specificity** | **Threshold** |
| --- | --- | --- | --- | --- | --- | --- | --- |
| 99.14 | 96.55 | 1090 | 100.00 | 1.00 | 1.00 | 1.00 | 487 |
| 99.14 | 96.55 | 1117 | 100.00 | 1.00 | 1.00 | 1.00 | 487 |
| 98.80 | 96.67 | 1224 | 89.14 | 0.78 | 0.89 | 0.89 | 423 |
| 99.51 | 98.97 | 1239 | 85.14 | 0.70 | 0.84 | 0.87 | 501 |
| 99.16 | 96.68 | 1297 | 100.00 | 1.00 | 1.00 | 1.00 | 481 |
| 99.19 | 96.94 | 1134404 | 100.00 | 1.00 | 1.00 | 1.00 | 492 |
| 99.15 | 97.19 | 142182 | 100.00 | 1.00 | 1.00 | 1.00 | 497 |
| 99.13 | 96.81 | 187144 | 100.00 | 1.00 | 1.00 | 1.00 | 512 |
| 99.06 | 97.50 | 32066 | 87.50 | 0.77 | 0.75 | 1.00 | 496 |
| 99.19 | 96.87 | 40117 | 100.00 | 1.00 | 1.00 | 1.00 | 500 |
| 99.17 | 96.74 | 48497 | 100.00 | 1.00 | 1.00 | 1.00 | 504 |
| 99.13 | 97.08 | 57723 | 100.00 | 1.00 | 1.00 | 1.00 | 485 |
| 99.14 | 96.56 | 65842 | 100.00 | 1.00 | 1.00 | 1.00 | 502 |
| 99.17 | 96.95 | 67812 | 100.00 | 1.00 | 1.00 | 1.00 | 488 |
| 99.13 | 96.94 | 67819 | 100.00 | 1.00 | 1.00 | 1.00 | 477 |
| 99.17 | 97.07 | 74201 | 94.74 | 0.89 | 0.95 | 0.95 | 489 |
| 99.22 | 97.22 | 976 | 96.94 | 0.94 | 0.94 | 1.00 | 492 |
| 99.14 | 97.13 | 256845 | 100.00 | 1.00 | 1.00 | 1.00 | 507 |
| 99.15 | 97.04 | 200795 | 94.44 | 0.89 | 1.00 | 0.89 | 504 |
| 99.15 | 97.00 | 200918 | 100.00 | 0.58 | 1.00 | 0.50 | 487 |
| 99.22 | 96.87 | 201174 | 91.26 | 0.83 | 0.98 | 0.85 | 495 |
| 99.16 | 97.21 | 203682 | 96.88 | 0.94 | 1.00 | 0.94 | 494 |
| 99.08 | 96.82 | 203691 | 100.00 | 1.00 | 1.00 | 1.00 | 510 |
| 99.11 | 96.92 | 408169 | 96.15 | 0.93 | 1.00 | 0.92 | 507 |
| 99.13 | 96.55 | 544448 | 100.00 | 1.00 | 1.00 | 1.00 | 475 |
| 99.11 | 97.13 | 640293 | 100.00 | 1.00 | 1.00 | 1.00 | 502 |
