# Supplementary material for: SCMBYK: prediction and characterization of bacterial tyrosine-kinases based on propensity scores of dipeptides
Source: BMC Bioinformatics. 2016 Dec 22;17(Suppl 19):514. doi: 10.1186/s12859-016-1371-4 (PMC5260027; doi:10.1186/s12859-016-1371-4)
Supplement: Additional file 3: Table S2. — The propensity scores and composition (%) of amino acids in BY-kinases. (DOCX 13 kb) [file 12859_2016_1371_MOESM3_ESM.docx]

Table S2. The propensity scores and composition (%) of amino acids in BY-kinases

| Amino acid | BY-kinase Score (Rank) | Composition of BY-kinases: A(%) | Composition of non-BY-kinases: B(%) | Composition difference: A-B(%) |
| --- | --- | --- | --- | --- |
| S-Ser | 593.63(1) | 7.57 | 5.67 | 1.89 |
| L-Leu | 571.30(2) | 11.38 | 9.76 | 1.62 |
| Q-Gln | 522.18(3) | 4.70 | 3.59 | 1.11 |
| R-Arg | 499.95(4) | 6.17 | 5.15 | 1.02 |
| T-Thr | 475.28(5) | 5.74 | 5.37 | 0.37 |
| V-Val | 472.78(6) | 7.61 | 7.08 | 0.53 |
| N-Asn | 462.13(7) | 4.27 | 3.91 | 0.36 |
| Y-Tyr | 438.55(8) | 3.12 | 3.09 | 0.03 |
| A-Ala | 433.15(9) | 9.47 | 9.10 | 0.36 |
| P-Pro | 431.60(10) | 4.18 | 4.06 | 0.12 |
| D-Asp | 405.00(11) | 5.39 | 5.83 | -0.44 |
| C-Cys | 390.98(12) | 0.38 | 0.84 | -0.47 |
| E-Glu | 383.73(13) | 6.34 | 6.77 | -0.43 |
| M-Met | 383.00(14) | 2.00 | 2.54 | -0.54 |
| I-Ile | 377.13(15) | 6.19 | 6.87 | -0.68 |
| W-Trp | 373.10(16) | 0.45 | 1.02 | -0.57 |
| K-Lys | 370.68(17) | 5.22 | 6.04 | -0.82 |
| H-His | 342.00(18) | 1.20 | 2.05 | -0.84 |
| F-Phe | 304.53(19) | 2.72 | 3.96 | -1.25 |
| G-Gly | 287.40(20) | 5.92 | 7.30 | -1.38 |
| R | 1.00 | 0.55 | 0.27 | 0.99 |
