# Supplementary material for: SCMBYK: prediction and characterization of bacterial tyrosine-kinases based on propensity scores of dipeptides
Source: BMC Bioinformatics. 2016 Dec 22;17(Suppl 19):514. doi: 10.1186/s12859-016-1371-4 (PMC5260027; doi:10.1186/s12859-016-1371-4)
Supplement: Additional file 4: Table S3. — The averaged a-helices contents. (DOCX 15 kb) [file 12859_2016_1371_MOESM4_ESM.docx]

Table S3. The averaged a-helices contents

|  | BY-kinase(%) | Non-BY-Kinase(%) | P-value* |
| --- | --- | --- | --- |
| SMOPA | 45.44±7.68 | 41.56±12.76 | <0.001 |
| NetSurfP | 48.13±9.29 | 42.05±19.92 | <0.001 |

* evaluating using t-test
